# Drug induced oral erythema multiforme: Case report

**DOI:** 10.1097/MD.0000000000022387

**Published:** 2021-04-30

**Authors:** Shaik Mohamed Asif, Shaik Mohamed Shamsudeen, Khalil Ibrahim Assiri, Hussain Mohammed Al Muburak, Sultan Mohammed Kaleem, Abdul Ahad Khan, Mansoor Shariff

**Affiliations:** a Department of Diagnostic Science and Oral Biology; b Department of Oral & Maxillofacial Surgery; c Department of Prosthetic Dentistry, College of Dentistry, King Khalid University, Abha, Kingdom of Saudi Arabia.

**Keywords:** drugs, oral erythema multiforme, ulceration

## Abstract

**Introduction:**

Drug induced oral erythema multiforme a rare clinical entity which involves only the lips and oral mucosa without skin involvement. These lesions are difficult in diagnosing with other oral ulcerative lesions with similar clinical manifestations.

**Patient concerns:**

This article presents 2 case reports of Oral erythema multiforme in which drugs were the precipitating factor. Its etiopathogenesis, differential diagnosis and treatment modalities of the disease is discussed.

**Diagnosis:**

Based on patient's complaints, drug history and clinical appearance, provisional diagnosis of drug induced erythema multiforme was considered.

**Intervention:**

For case 1, patient was instructed to discontinue usage of drug and prescribed systemic steroid (Prednisolone 10 mg/d) for a week along with germicidal drugs to prevent secondary infection. Medication was tapered to 5 mg/d after first week.

For case 2, patient was instructed to discontinue the drug and systemic steroid prednisolone 20 mg /d for 1 week with tapering dose of 10 mg/d for the second week was administered.

**Outcome:**

For case 1 and case 2 healing of the lesions were evident on third week of follow up.

**Conclusion:**

Medications should be taken under medical supervision. Over the counter drugs might lead to allergic reactions like drug induced oral erythema multiforme, which is a rare variant and needs to be differentiate from other oral ulcerative lesion for prompt management and follow-up.

## 1 Introduction

Erythema multiforme (EM) is an acute self-limiting hypersensitive muco cutaneous lesion with varied etiologies. It is classified as spectrum of disorders with Erythema multiforme minor, Erythema multiforme major, Steven Johnson Syndrome and toxic epidermal necrosis considering Erythema multiforme minor the mildest and toxic epidermal necrosis the most severe form.^[1]^ Herpes virus infection is considered to be involved more than 90% of cases. Drug associated EM is rare and reported to be less than 10%.^[2]^ EM affects most commonly teenagers and young adults with more predilection to males.^[3]^ Oral lesions are seen on the lips and buccal mucosa which appear as erythematous macules, and bloody encrustations involving lips. Isolated oral lesions are rare entity which makes diagnosis in question. This rare variant has been considered as Oral erythema multiforme.^[4,5]^ Here in we present 2 case reports of oral erythema multiforme which were induced by drugs. Its etiology, pathogenesis and diagnostic criteria have been emphasized.

## 2 Case report

### 2.1 Case-1

**Figure 1:**
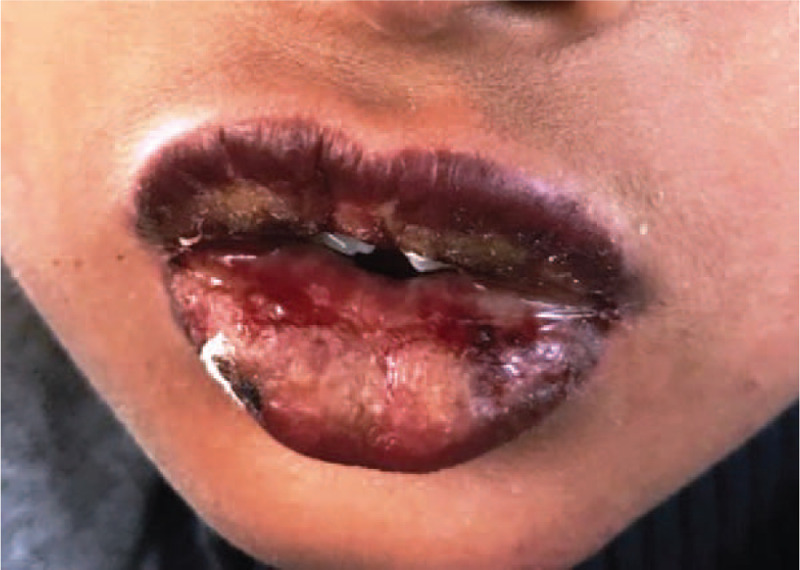
Ulcerative encrusted lesions on lips.

**Figure 2:**
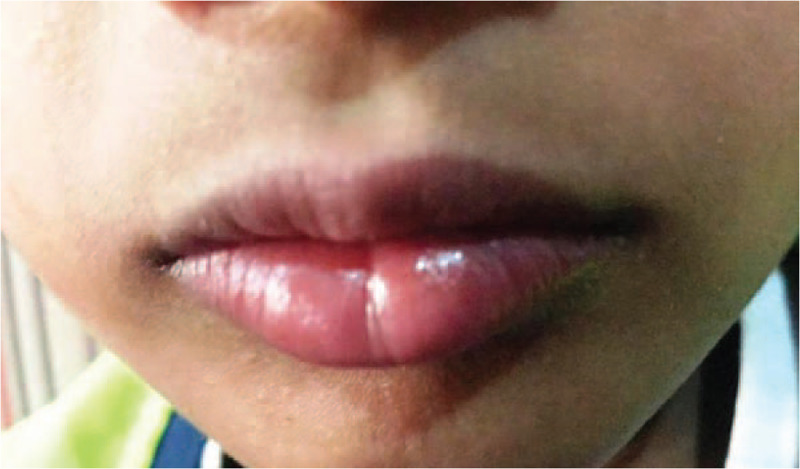
Healed lesion after 3 week follow up.

A 13 year old male reported to department of Oral Medicine with a complaint of burning sensation in oral cavity since 2 days. Initially patient experienced burning sensation an hour after administration of over counter drug (Diclofenac sodium). Burning sensation became severe after administration of the second dose. There was no history of fever after administration of drug. History of difficulty in swallowing and disturbed sleep was elicited due to burning sensation. No skin or ocular lesions were evident. On examination multiple ulcers on vermilion border of upper and lower lips were observed (Fig. 1). Ulcers were encrusted showing varying size of 1 to 2 mm. Based on positive drug history and evidence of lesions, provisional diagnosis of drug induced erythema multiforme was given. Patient was instructed to discontinue usage of drug and prescribed systemic steroid (Prednisolone 10 mg/day) for a week along with germicidal drugs to prevent secondary infection. Medication was tapered to 5 mg/d after first week. Lesion completely resolved on third week of follow up (Fig. 2).

### 2.2 Case -2

**Figure 3:**
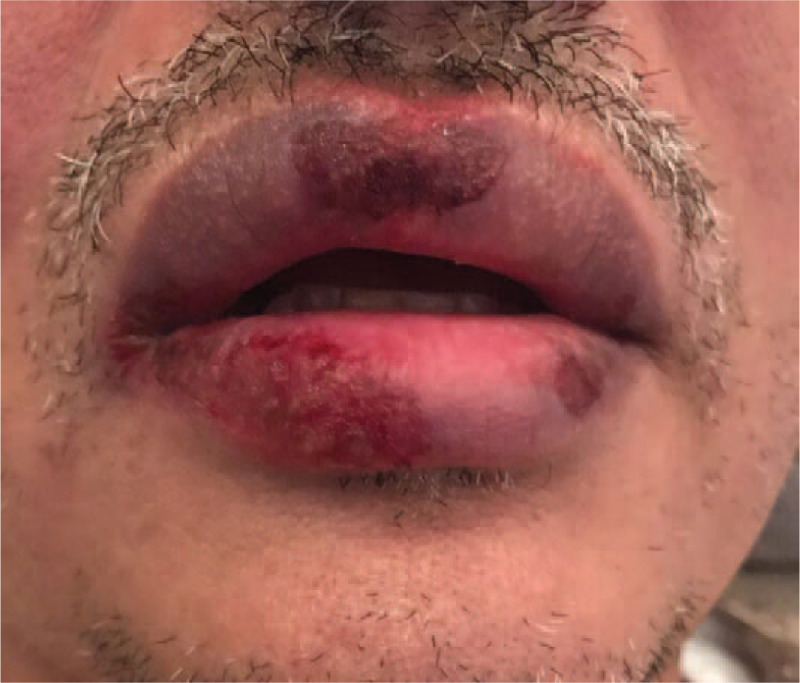
Encrusted ulcers on lips.

**Figure 4:**
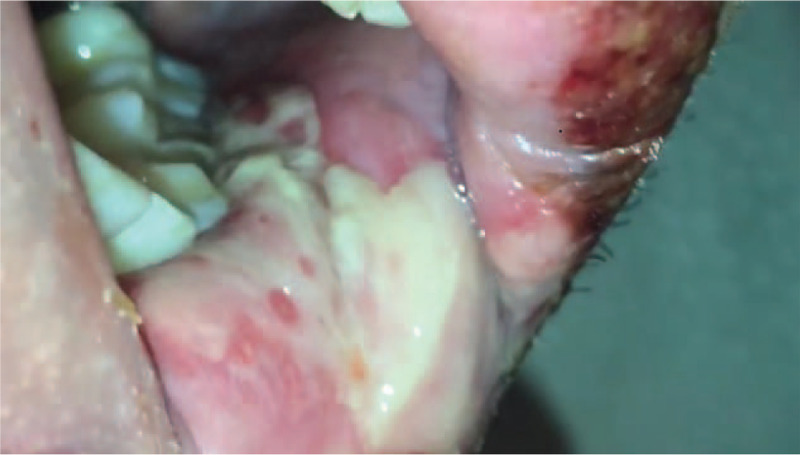
Ulcerative lesion on left buccal mucosa.

**Figure 5:**
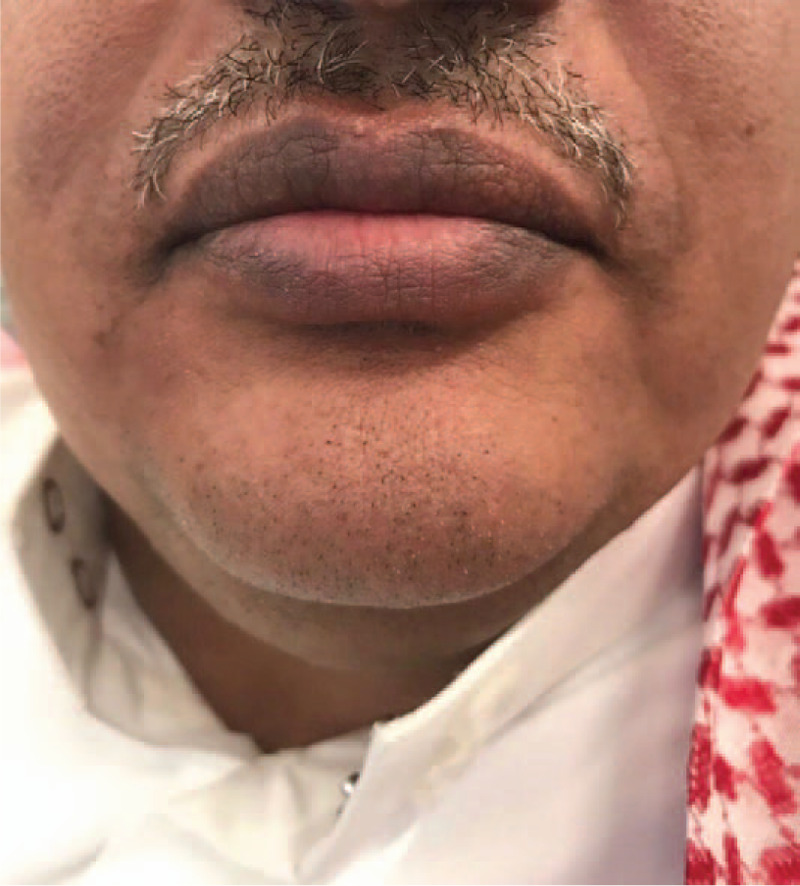
Healed lesion after 3 weeks of follow up.

**Figure 6:**
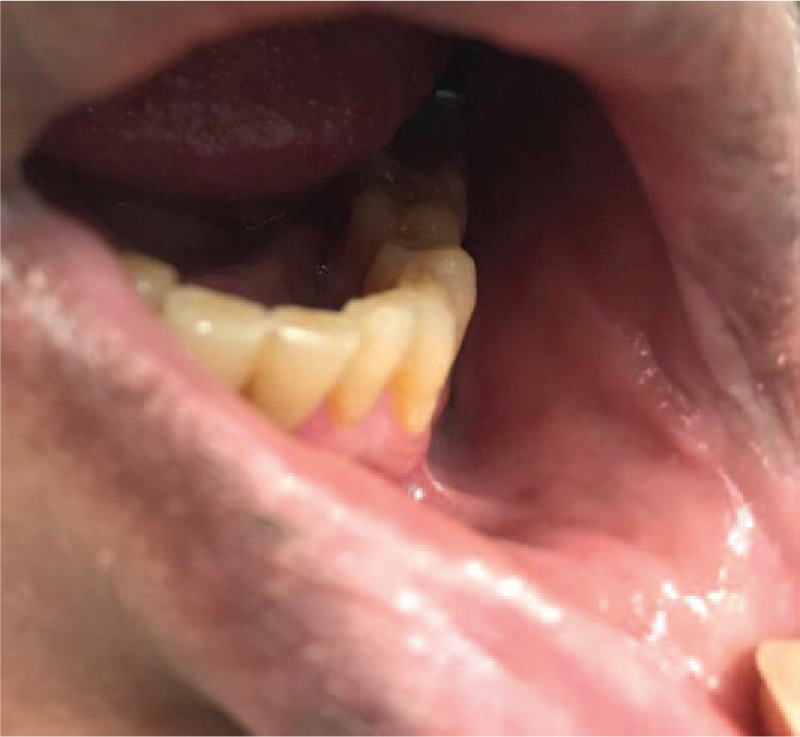
Healed lesion after 3 weeks of follow up.

A 42 year old male reported to Department of Oral Medicine with chief complaint of burning sensation in oral cavity since 1 day. Burning sensation was sudden in onset due to administration of over counter medication (Paracetamol 500 mg) for sore throat. There was no history of fever. Burning sensation became severe after subsequent dose. Patient gave history of difficulty in swallowing. Multiple blood tinged encrusted ulcers were seen on vermilion border of both lips which were tender on palpation and bleeding on touch (Fig. 3). Intra oral examination revealed ulceration on patient's left buccal mucosa with yellowish slough which was tender on palpation (Fig. 4). Similar lesions were noticed on oro pharyngeal region. Based on patient's complain, drug history and clinical appearance, provisional diagnosis of drug induced erythema multiforme was considered. Patient was instructed to discontinue the drug and systemic steroid prednisolone 20 mg /d for 1 week with tapering dose of 10 mg/d for the second week was administered. Healing of the lesions were evident on third week of follow up. (Figs. 5 and 6).

## 3 Discussion

Drugs are double edged sword, which gives beneficial results and can also cause adverse reaction in certain conditions. Adverse drug reaction can manifest as many forms like Erythema multiforme, fixed drug eruption and anaphylactic reactions. Erythema multiforme was first reported in literature by Bateman and Bulkey in 1846 followed by Hebra^[6]^ in 1866 who described as erythema exudative multiforme. In 1922 Stevens and Johnsons reported severe form of erythema multiforme with involvement of oral and conjunctival mucous membrane along with skin lesions.^[7]^ In 1968, Kenneth described an inflammatory oral disorder which resembled to that of erythema multiforme without any skin involvement.^[8]^ EM due to drugs is rare, most common drugs which induce reactions are non-steroidal anti-inflammatory drugs and antibiotics Very few cases of EM have been reported with the ingestion of paracetamol and diclofenac sodium. Database showed 2.06% of cases of EM were due to diclofenac sodium and 6.17% of cases were due to ingestion of paracetamol.^[9]^ In our cases, manifestation of the lesion appeared after intake of initial drug. Patient had not been exposed to any kind of infection or allergic to any food additives. Hence with this temporal occurrence of drug intake and appearance of the lesion, it was considered that etiological agent was drug in both patients. The probability of adverse drug reaction was 5 according to Naranjo scale.^[10]^ Drug induced EM express tumor necrosis factor alpha instead of interferon–gamma. Drug metabolism is altered and directed towards cytochrome p450- metabolite pathway resulting in production of reactive and toxic metabolites. Tissue damage is mainly due to apoptosis and not by inflammatory response. Oral EM is a distinct but less well recognized variant of EM. Very few cases have been reported in the literature with this form.^[11]^ It has been reported that primary attacks of oral EM are confined to oral mucosa without skin involvement. Subsequent attacks can produce more severe form of EM involving the skin.^[12]^ Oral mucosa is the commonest site with labial mucosa, buccal mucosa and lips involving in 70% of cases. In our patient, ulceration was confined to lips and buccal mucosa. Classical target lesions were not seen on the skin. In considering differential diagnosis, most common is apthous ulcer which occurs commonly on the lining mucosa. They are round or oval ulcers with yellowish gray membrane surrounded by erythematous halo. Herpes simplex infections are seen more on attached mucosa like gingiva, palate and lips with regular margins and small in size.^[11]^ In our case there were no such lesion seen on the attached mucosa. Recurrent herpes labialis can affect lips, but onset is viral and our patient does not have any prodromal symptoms with clinical features not correlate to that of viral infection. Lesions like pemphigus are seen on gingiva resembling desquamative gingivitis. An important clinical sign noted in pemphigus is nikolsky sign^[11]^ which was negative in our patient. Fixed drug eruption occurs due to hypersensitivity reaction to drugs characterized by skin lesions that recur at same anatomical location upon repeated exposures to drug.^[13]^ Diagnosis of EM mainly rely's on clinic onset, positive triggering factor and clinical appearance. Histopathology of EM will be nonspecific and biopsy can be obtained only in vesicular stage of lesion. In our case, ulcerative stage of the lesion restricted biopsy. Treatment of EM is based on identification and removal of triggering factors. These lesions usually respond to corticosteroid, topical steroids can be started in case of minor lesion and systemic steroids for severe conditions for a period of 1 week with tapering dose. In our patients offending drug was discontinued and were prescribed systemic steroids for a week and later tapered. Lesion healed completely in 10 days without any scar formation.

## 4 Conclusion

Drug induced Oral erythema multiforme is a rare variant and needs to differentiate from other oral ulcerative lesion for prompt management and follow-up. Treatment of oral EM is symptomatic and involves treating the underlying causes. However, use of short course of systemic prednisone have been reported to very effective in controlling lesions as supported by the dramatic response in our patients.

## Author contributions

SMA &SMS performed the initial examination, patient assessment and prescribed medication for the patient. SMS, KIA, SMK, recalled and followed up patient after 3 weeks SMK, AAK, HAM, MS reviewed the available literature and drafted the manuscript. AAK & MS reviewed the literature and corrected the drafted manuscript. All authors have read and approved the manuscript.

## Corrections

The titles Assistant Professor were originally part of all three affiliations and have since been removed to follow journal style.
